# Evaluation of roadside air quality using deep learning models after the application of the diesel vehicle policy (Euro 6)

**DOI:** 10.1038/s41598-022-24886-z

**Published:** 2022-12-01

**Authors:** Hyemin Hwang, Sung Rak Choi, Jae Young Lee

**Affiliations:** 1 Environmental Engineering Department, Ajou University, Suwon, 16499 Korea; 2 Environmental and Safety Engineering Department, Ajou University, Suwon, 16499 Korea

**Keywords:** Environmental sciences, Environmental impact

## Abstract

Euro 6 is the latest vehicle emission standards for pollutants such as CO, NO_2_ and PM, that all new vehicles must comply, and it was introduced in September 2015 in South Korea. This study examined the effect of Euro 6 by comparing the measured pollutant concentrations after 2016 (Euro 6–era) to the estimated concentrations without Euro 6. The concentration without Euro 6 was estimated by first modeling the air quality using various environmental factors related to diesel vehicles, meteorological conditions, temporal information such as date and precursors in 2002–2015 (pre–Euro 6–era), and then applying the model to predict the concentration after 2016. In this study, we used both recurrent neural network (RNN) and random forest (RF) algorithms to model the air quality and showed that RNN can achieve higher R^2^ (0.634 ~ 0.759 depending on pollutants) than RF, making it more suitable for air quality modeling. According to our results, the measured concentrations during 2016–2019 were lower than the concentrations predicted using RNN by − 1.2%, − 3.4%, and − 4.8% for CO, NO_2_ and PM_10_. Such reduction can be attributed to the result of Euro 6.

## Introduction

Vehicular exhaust is a major source of pollutants that affects regional and global urban air quality. The European Environment Agency reported that the road transport sector contributes majorly to nitrogen oxides (NO_x_) and black carbon^1^. Similarly, in Korea, the transport sector accounts for the largest contribution of NO_x_ emissions^2^. Especially, diesel engines are well known for high NO_x_ emissions^3^. In addition, diesel vehicles also emit other air pollutants, such as particulate matter (PM), volatile organic carbons, and polyaromatic hydrocarbons^4^. All of these pollutants are known to have adverse effects on human health^5^. Acute effects of exposure to diesel exhaust include irritation in the nose and eyes, lung function alterations, breathing pattern alterations, headache, fatigue, and nausea. Chronic exposure is associated with coughing, sputum production, and decreased lung functions^6^. Additionally, diesel exhaust directly affects ozone formation^7^.

The European Union has established automobile emission standards for NO_x_, dust, and CO. These standards, collectively referred to as Euro emission standards, have been periodically reinforced^8^. In Europe, Euro 1 was established in 1992, and the latest Euro 6 standards have been used since 2013. To meet these air pollutant emission regulations, various engine technologies have been developed. For example, selective catalytic reduction (SCR) devices^9^ converted NO_x_ from the exhaust gas into nitrogen (N_2_) and water (H_2_O) by injecting a reductant such as ammonia (NH_3_). Diesel particulate filters (DPFs) reduce particulate matters from the exhaust gas of a diesel engine^10^. Exhaust gas recirculation (EGR)^11^ is a method that can effectively reduce NOx emitted to the atmosphere. What EGR does is recirculating a portion of the exhaust gas back to the engine and mixing it with the atmospheric air in the combustion chamber. This reduces the concentration of O_2_ in the chamber, which in turn reduces the combustion of fuel and the peak combustion temperature, thereby reducing the generation of NOx from the process^12^. Generally, a combination of above technologies is used to meet the stringent emission control standards in modern diesel cars^13^.

To control air pollutant emissions from diesel vehicles, the Korean government has also implemented various policies. In Korea, Euro 1 was first introduced in 1994, and the Euro 6 standards were introduced in September 2015 to limit automobile exhaust gas emissions. In the context of Euro 6, all new diesel cars were mandated to install SCR to reduce NO_x_. In addition, Korea implemented policies such as providing subsidies for DPF attachments and early scrapping of high-emission diesel cars (since 2005)^14^, prohibiting old diesel vehicles from entering the metropolitan area from December to March (since December 2019), and implementing a vehicle emission rating system (since April 2020)^15^.

Previous researchers have reported that the Euro emission standards effectively reduced air pollution from automobile emissions^16–22^. In Spain, vehicular CO and PM emissions have considerably reduced due to Euro 4 and 5 vehicular emission standards ^16^. In Paris and London, NO_2_ and PM concentrations decreased owing to Euro 5^17^. Grigoratos et al. tested five Euro 6 heavy duty vehicles (HDVs) on-road under typical driving conditions and showed that all tested vehicles emitted less pollutants compared to HDVs with previous technologies^18^. Kim et al. investigated the NO_x_ emissions of 132 certified Euro 6 light duty diesel vehicles in Korea, and they analyzed the installed after-treatment devices and the vehicles’ control strategies^19^. Ko et al.^20^ reported low NO_x_ emissions of Euro 6 vehicles under actual driving conditions, regardless of the total vehicle weight. Despite many previous studies, the effects of Euro 6 policies on the air pollutant concentration in the roadside air quality in Korea have not been studied. Therefore, it is necessary to study the roadside air quality in Korea after the introduction of Euro 6 by comparing the air quality before and after the introduction.

Recently, air quality has been predicted using a combination of different artificial intelligence technologies^23–26^. Jo et al*.* compared the performance of air quality prediction systems using deep neural network and long short-term memory (LSTM) models, which are used for time-series data-based predictions^23^. Athira et al. predicted air quality using recurrent neural network (RNN), LSTM, and gated recurrent unit (GRU) based on pollution and meteorological time-series AirNet data^24^. Tao et al. predicted air pollution by presenting a convolution-based bidirectional GRU method based on 1-D convolutional neural networks and bidirectional GRU neural networks^25^. Feng et al. explained the causes of winter PM_10_ fluctuations in nine Chinese cities using random forest (RF) and RNN^26^.

In this study, the association between air quality and the diesel vehicles was modeled using RNN and RF. RNN is a type of artificial neural network that is suitable to process sequential data^27,28^. It has a structure that can feedback outputs into inputs and has an internal memory that can remember important things from previous inputs. This structure makes it suitable for handling the dynamics of sequential data^28^. RF is an ensemble of many decision trees, and it is known as one of the best performing machine learning algorithms for various classification and regression problems^29^. The high model accuracy and the accurate prediction results can be achieved with RF by minimizing overfitting through various techniques^30^. Since RNN and RF are both very popular algorithms for great modeling performance, this study decided to use both algorithms to model the air quality.

Using RNN and RF prediction models, this study aimed to determine how the Euro 6 diesel vehicle policy influenced the roadside air quality in Korea. Specifically, we evaluated the importance of various environmental factors such as the diesel vehicle registration number and meteorological conditions in determining the concentrations of each of four pollutants (CO, NO_2_, O_3_, PM_10_) during 2002–2015, and then we used the information to eliminate the features with low importance. Then the air quality during 2002–2015 was modeled using the selected features based on both RF and RNN. Since the most of the modeling period is before the introduction of Euro 6 (introduced in Sep. 2015), these models didn’t include the effect of Euro 6. Finally, we used these models to predict the air pollutant concentration for Euro 6–era from 2016 to 2020 and calculated any systematic deviations between the predicted and measured concentrations to assess the effect of Euro 6 in Korea. To the best of our knowledge, this is the first study model the air pollutant concentrations using RF and RNN and examined the effect of Euro 6 in Korea.

## Method

### Data collection and pre-processing

Daily concentrations of CO, NO_2_, O_3_, PM_10_, and SO_2_ measured from 49 roadside air quality monitoring stations (AQMS) in Korea during 2002–2020 were collected from the Korea Environment Corporation^31^. The data from 49 stations were averaged to obtain the country-wide overall roadside air quality in Korea for modeling the impact of vehicle emissions on the air pollutant concentrations.

Daily meteorological data measured from 100 monitoring stations in Korea during 2002–2020 were collected from the Korea Meteorological Administration^32^. The data include temperature, precipitation, relative humidity, insolation, wind direction, and wind speed, and the data from all stations were averaged to obtain the country-wide overall meteorological factors for the modeling. The behavior of the wind was initially expressed as a vector using wind direction and wind speed. For the averaging of the vectors measured from multiple stations, we converted the wind vectors into the north–south (NS) and east–west (EW) components by projecting the vectors on to those two axes. This can be expressed as the following equations.

1 $$ {\text{Wind NS}} = {\text{wind speed}} \times {\text{cos}}\left( {\text{wind direction}} \right) $$

2 $$ {\text{Wind EW}} = {\text{wind speed}} \times {\text{sin}}\left( {\text{wind direction}} \right) $$

These overall north–south and east–west winds are the factors that can be useful for modeling any possible long-range transport of air pollutants from nearby countries^33^.

Monthly diesel vehicle registration data during 2002–2020 were provided by the Ministry of Land, Infrastructure, and Transport^2^. In order to model the air quality on a daily basis using factors such as meteorological data and the number of diesel cars, the monthly registration data were linearly interpolated to obtain the daily data for the modeling.

All numerical variables were pre-processed using min–max normalization^34^ as expressed by

3 $$ {\text{Normalized}} = \frac{{{\text{Original}} - {\text{Min}}}}{{{\text{Max}} - {\text{Min}}}} $$

The day of the week, a categorical variable, was pre-processed using one-hot encoding^35^.

### Feature selection

In this study, we used four categories of factors to model the air pollutant concentrations. The first was the number of registered diesel vehicles. The second was the meteorological factors, including temperature, relative humidity, insolation, precipitation, wind NS and wind EW. Meteorological factors were important, since they could influence the processes of diffusion, generation, and removal of air pollutants^36^. The third was the temporal factors. The day of the year, day of the week, and date were included in this category to model the weekly, seasonal and long-term variations in the concentrations of air pollutants. The fourth was the precursor factors. It is known that the presence of gaseous pollutants (CO, NO_2_, and SO_2_) can serve as the precursors to facilitate the generation of O_3_ and PM_10_. Figure S1 presented a graphical explanation of such secondary formation^37,38^. Therefore, we used these factors for modeling the concentrations of O_3_ and PM_10_.

Among the four categories of factors, we selected the final factors to be provided in modeling the pollutant concentrations based on the feature importance analysis^30,39^. The feature importance analysis evaluated how important each feature is in explaining the air pollutant concentrations. By removing the features with low importance, we can reduce the possibility of overfitting. To perform the feature importance analysis, the “GridSearchCV” function of “sklearn” package of Python (ver. 3.10.2) was used^40^.

### Modeling using RF and RNN in pre–Euro 6–era (2002–2015)

Air pollutant concentrations were modeled based on RF and RNN using the selected features from 2002 to 2015, when Euro 6 has not been applied yet. Due to the choice of the period, the effect of Euro 6 was not included in the modeling. RF and RNN were two of the most popular machine learning algorithms and the relative performance of those two algorithms depends on the application and the size of dataset^41^. Therefore, we decided that it was worth trying both RF and RNN for modeling.

We used a validation technique for robust modeling, where 70% of the modeling data were randomly selected as a training set and 30% as a validation set. The validation set were used to evaluate the performance of the training, and the iteration in training stopped when the loss function value on the validation set started to increase owing to overfitting^28^.

The “sklearn” package of Python was used for RF. The main parameter in the RF model was “best_estimator_” of the “GridSearchCV” function, and the optimal value for each variable was set automatically^40^. The model parameters used in the model are summarized in Table S1. The root mean square error was used as the loss function, and R^2^ was checked to analyze its accuracy^42^.

The “keras^43^” and “tensorflow^44^” packages of Python were used for RNN. The main parameters of RNN are listed in Table S2. For the RNN layer, “simpleRNN,” the most basic RNN, was used^45^, and 64 neurons were added to the layer. An activation function, “tanh,” was used to convert the sum of the input signals into an output signal. Compared with other functions, the “tanh” function is zero-centered and can be used for optimization^28^. Using the Adam optimizer^46^, the loss function was calculated based on the mean absolute error and the learning rate was set to 0.001. Figures S2–S5 show the training and validation losses according to the RNN epochs.

### Prediction using the models for Euro 6–era (2016–2020)

The RF and RNN models trained using the data from pre–Euro 6–era (2002–2015) were used to predict the air pollutant concentrations for Euro 6–era (2016–2020). The difference between the predicted and measured average concentrations during 2016–2020 was calculated to see any systematic deviations between the model and the reality, part of which may be due to the effect of Euro 6. The equation used to calculate the deviation is given by

4 $$ {\text{Deviation}}\left( {\text{\% }} \right) = \frac{{{\text{Observed}} - {\text{Predicted}}}}{{{\text{Observed}}}} \times 100. $$

## Results

### Selection of features that affect pollutant concentration

Figure S6 shows that number of diesel vehicle registrations in Korea increased steadily during 2002–2020 and CO, NO_2_, O_3_, and PM_10_ showed periodicity due to seasonal fluctuations. CO and NO_2_ tended to decrease, while O_3_ tended to increase during 2002–2020. The increase in O_3_ may be due to increased anthropogenic precursor emissions and long-term changes in meteorology. In particular, rising global temperatures may cause ozone concentrations to increase^47^. The strong spike in PM_10_ could be attributed to long-range transport^48^. Because strong spikes caused by long-range transport increase uncertainty in the concentration prediction, PM_10_ trends were analyzed after removing outliers. Figure S7 shows the trend of the annual number of diesel vehicles per diesel vehicle type. Except passenger cars, the number of other vehicle types did not change significantly during 2002–2020. However, the proportion of passenger cars was 20.5% in 2002 among all diesel vehicles; subsequently, this ratio steadily increased, and in 2020, passenger cars accounted for the largest percentage (58.6%) among all domestic diesel vehicles. Overall, passenger cars and trucks accounted for most number of diesel vehicle registrations.

**Figure 1:**
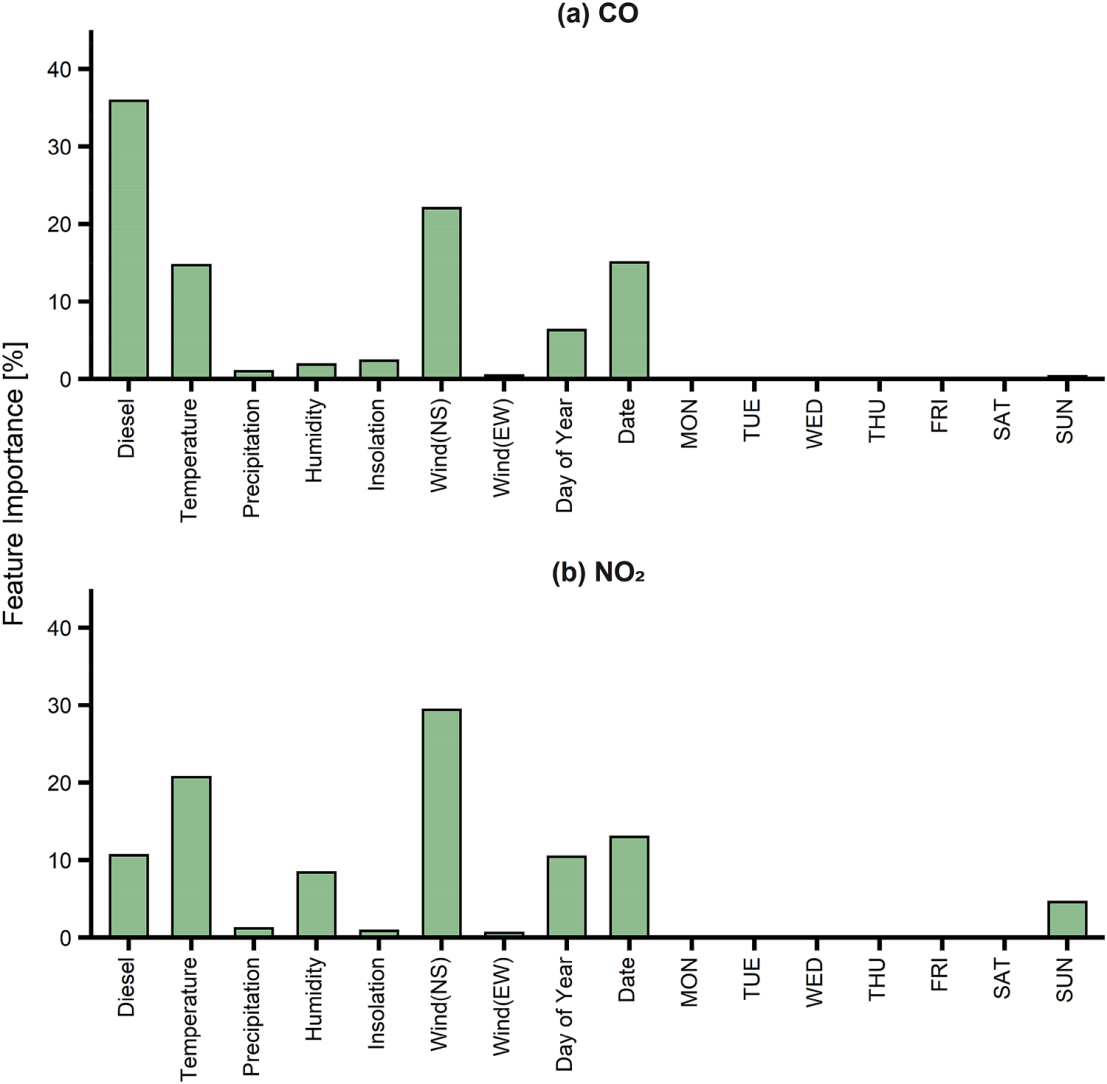
Feature importance of (**a**) CO and (**b**) NO_2_.

**Figure 2:**
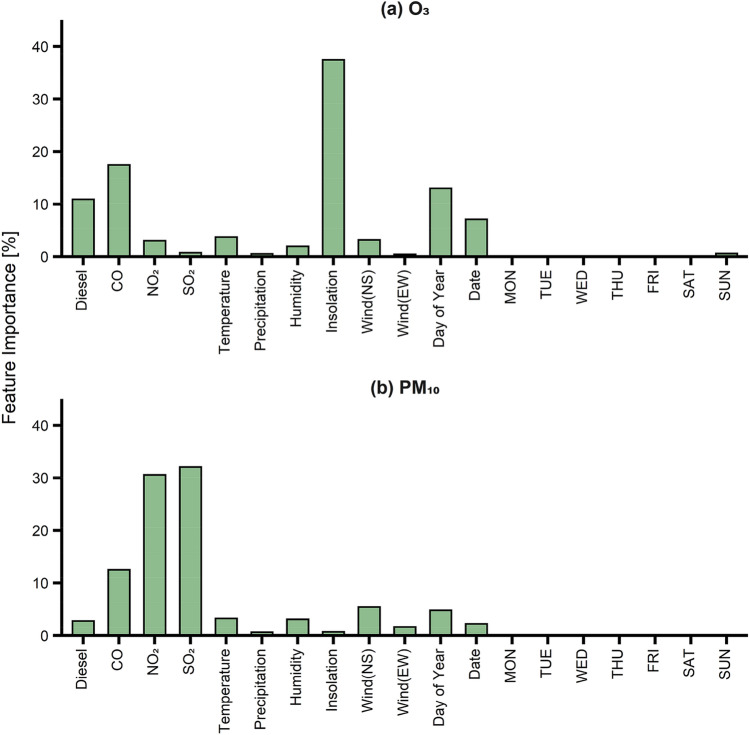
Feature importance of (**a**) O_3_ and (**b**) PM_10_.

Feature importance analysis was performed based on RF to select the final input factors for modeling. Table S3 shows the results of the feature importance analysis. Figure 1 shows the results of the feature importance analysis for CO and NO_2_. The number of diesel vehicles, temperature, north–south wind, and date have a major influence on CO and NO_2_. Figure 2 shows the results of the feature importance analysis for O_3_ and PM_10_. The influence of insolation was the largest (37.4%) for O_3_. Insolation is involved in the photochemical reaction of O_3_, and is more important than the presence of gases, such as CO, NO_2_, and SO_2_^49^. PM_10_ was dominantly influenced by the concentrations of CO, NO_2_, and SO_2_. This is because PM_10_ could be produced not only from primary emissions, but also from gas-to-particle conversion of gaseous pollutant emitted from roadside air^50^ (see Figure S1 for the graphical illustration of the process). Among various features, the day of the week showed only a small influence on the air pollutant concentrations. Therefore, the day of the week was not selected as the final input data in the RF and RNN models.

### Modeling and prediction using RF and RNN models

The air pollutant concentrations were modeled based on RF and RNN algorithms using data from 2002 to 2015. Table S4 shows the R^2^ and RMSE of the models on the validation set (2002–2015) for each air pollutant. As can be seen, RF shows R^2^ of 0.44 ~ 0.635 and RNN shows R^2^ of 0.634 ~ 0.759 depending on the pollutants, suggesting that the performance of the RNN model was better than that of the RF model.

**Figure 3:**
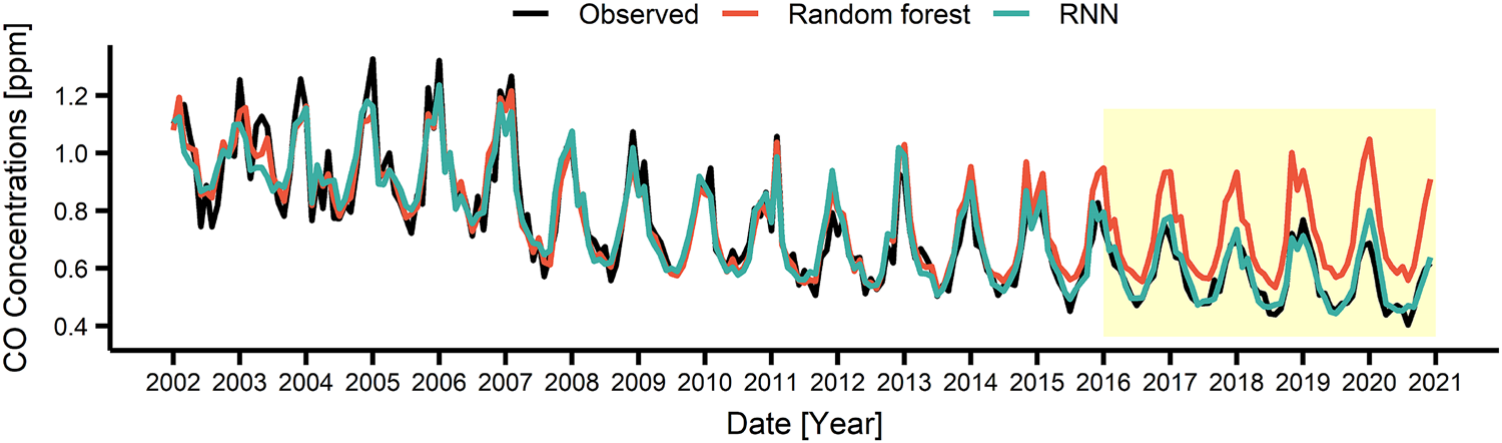
Monthly concentrations of observed and predicted values by using recurrent neural network (RNN) and random forest (RF) for CO. The area shaded in yellow showed the period when the Euro 6 was applied.

**Figure 4:**
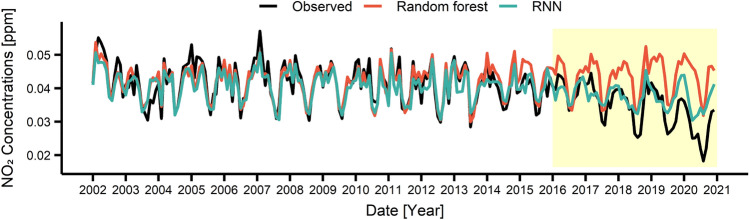
Monthly concentrations of observed and predicted values by using RNN and RF for NO_2_. The area shaded in yellow showed the period when the Euro 6 was applied.

**Figure 5:**
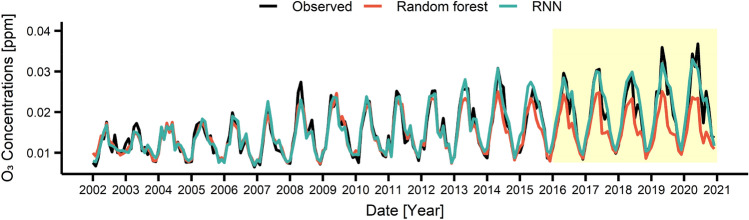
Monthly concentrations of observed and predicted values by using RNN and RF for O_3_. The area shaded in yellow showed the period when the Euro 6 was applied.

**Figure 6:**
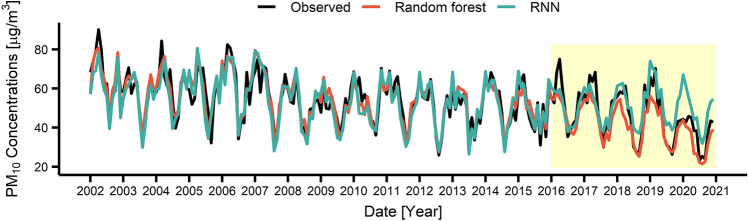
Monthly concentrations of observed and predicted values by using RNN and RF for PM_10_. The area shaded in yellow showed the period when the Euro 6 was applied.

These RF and RNN models were used to predict the air pollutant concentrations during 2016–2020. Figures 3, 4, 5, 6 present the air pollutant concentrations modeled (2002–2015) and predicted (2016–2020) by the RF (in red) and RNN (in blue) models along with the observed concentrations (in black). The prediction period (2016–2020), coincided with the Euro 6 period, was depicted as yellow box in Figs. 3, 4, 5, 6. Note that the modeled curves agreed well with the observed curve during 2002–2015 and started to deviate from the observation since 2016. Table 1 lists the deviations between the modeled and measured average concentrations for 2002–2015 (before Euro 6) and between the predicted and measured average concentrations for 2016–2020 (after Euro 6). Note that the deviation during modeling period is relatively small with  − 1.3 ~ 6.7 for RF and – 0.3 ~ 2.4 for RNN depending on the pollutants, while the deviation during the prediction period is larger with – 26.6 ~ 20.0 for RF and  – 8.8 ~  – 2.1 for RNN. This result also suggested that the performance of the RNN model was better than that of the RF model. Particularly, the RNN model succeeded in predicting the long-term decrease trend for CO, NO_2_ and PM_10_ as well as the long-term increase trend for O_3_ during 2016–2020. The directions of the change were correct for all pollutants, but the magnitudes of the change were predicted to be less. However, the RF model failed to predict the long-term trends for both direction and magnitude.

**Table 1:** Difference between the predicted value of the model and the actual observed value according to air pollutants.

Period	Model	CO		NO_2_		O_3_		PM_10_	
		Conc. (ppm)	Deviation (%)	Conc. (ppm)	Deviation (%)	Conc. (ppm)	Deviation (%)	Conc. (μg/m^3^)	Deviation (%)
2002–2015	Observed	0.794		0.042		0.015		54.97	
	RF	0.804	− 1.3	0.042	0	0.014	6.7	55.34	− 0.7
	RNN	0.796	− 0.3	0.041	2.4	0.015	0	53.76	2.2
2016–2020	Observed	0.564		0.035		0.020		46.73	
	RF	0.714	− 26.6	0.044	− 25.7	0.016	20.0	41.68	10.8
	RNN	0.576	− 2.1	0.038	− 8.6	0.021	− 5.0	50.84	− 8.8

*RF* random forest, *RNN* recurrent neural network.

For the detailed analysis, Tables S5–S8 summarized observed, modeled and predicted yearly concentrations of each pollutant. As can be seen, the observed concentrations of CO, NO_2_, PM_10_ dramatically reduced in 2020, and the deviation between the observation and the RNN prediction in 2020 becomes − 6.2%, − 28.6%, and − 29.3% for CO, NO_2_, PM_10_. This can be mostly attributed to the lockdown measures applied during COVID-19 pandemic^51,52^. Except for 2020, the further decrease in the concentrations of air pollutants compared to the RNN prediction may be attributable to the environmental policies such as Euro 6.

## Discussion

This study modeled the air pollutant concentrations based on RF and RNN models with the diesel car registration information and various environmental factors in 2002–2015. Then, we predicted the air quality in 2016–2022 using the models. Finally, we calculated any systematic deviations between the predicted and observed concentrations in order to assess the effects of various regulations applied after 2015–2016 in South Korea. The high performance of the model trained in 2002–2015 could predict the air pollutant concentration accurately. Therefore, a difference between the concentration predicted by RNN and actual concentrations during 2016–2020 suggested the result of new policies introduced after the modeling period. Any policies that have been applied during 2002–2015 (e.g. early scrapping of high-emission diesel cars applied since 2005) were assumed to be captured in the modeling and not to contribute to the systematic deviations. Such policies applied after 2015–2016 include applying Euro 6 standards for new cars (since September 2015), and prohibiting old diesel vehicles from entering the metropolitan area during the cold season (since December 2019). According to our results, the measured concentrations were lower than the concentrations predicted using RNN by  – 1.2%, – 3.4%, and – 4.8% for CO, NO_2_ and PM_10_ during 2016–2019. Year 2020 was eliminated to rule out the effects of seasonal banning of old diesel cars and COVID-19 (both happened since December 2019). Since we eliminated the effects of other known policies, these reductions are likely to be originated from Euro 6.

This study aimed to investigate the air quality improvement associated with Euro 6 by comparing the measured concentrations with the ones predicted using a model constructed in pre–Euro 6–era. The methodology used in this study has a few limitations. First, the deviation of the observed from the predicted can stem not only from Euro 6 but also from many other differences between 2002 and 2015 and 2016–2020. In this study, we ruled out the effects of other policies and COVID-19, but there can be a yet another effect that hasn’t been ruled out. Any of those effects could make us overestimate the effect of Euro 6. Second, the diesel-car emissions depended on the mileage driven by the cars not on the number of vehicles. However, due to the availability of the data, we used the number of diesel cars instead. Third, we used factors related to diesel cars, meteorology, time, and precursors to model the air quality. However, other factors such as those related to industrial and non-industrial emissions were not included in the modeling due to the lack of the data. Providing such confounding factors might have improved the accuracy of the modeling. All of these limitations were originated from the lack of proper data and can be solved by collecting and building them. This work and the corresponding analysis were left for future work.

## Conclusions

The present study is the first to model the roadside air quality in South Korea using deep learning algorithms (RNN and RF) and to investigate the improvements in air quality after the introduction of Euro 6. To model the air quality, we prepared data on various environmental factors and performed feature analysis to select the final factors for modeling. As a result, we showed that the performance of the RNN model was better than that of the RF model in terms of R^2^. By comparing the measured concentrations with the ones predicted using RNN, we showed that Euro 6 contributed the pollutant reduction by − 1.2%, − 3.4%, and − 4.8% for CO, NO_2_ and PM_10_. These results may contribute to future policy making related to diesel vehicles, and the methodology used in this study can be applicable in assessing the effect of other policies.

## Supplementary Information

Supplementary Information.

## Data Availability

The datasets used and/or analyzed during the current study are available from the corresponding author on reasonable request.
