# Supplementary material for: Evaluation of roadside air quality using deep learning models after the application of the diesel vehicle policy (Euro 6)
Source: Sci Rep. 2022 Dec 1;12:20769. doi: 10.1038/s41598-022-24886-z (PMC9714413; doi:10.1038/s41598-022-24886-z)
Supplement: Supplementary file 1 — Supplementary Information. [file 41598_2022_24886_MOESM1_ESM.docx]

**Evaluation of Roadside Air Quality using Deep Learning Models after the Application of the Diesel Vehicle Policy (Euro 6)**

Hyemin Hwang^1^, Sung Rak Choi ^2^ and Jae Young Lee^2, *^

1. Environmental Engineering Department, Ajou University, Suwon 16499, Korea

2. Environmental and Safety Engineering Department, Ajou University, Suwon 16499, Korea

* Corresponding author: jaeylee@ajou.ac.kr

**
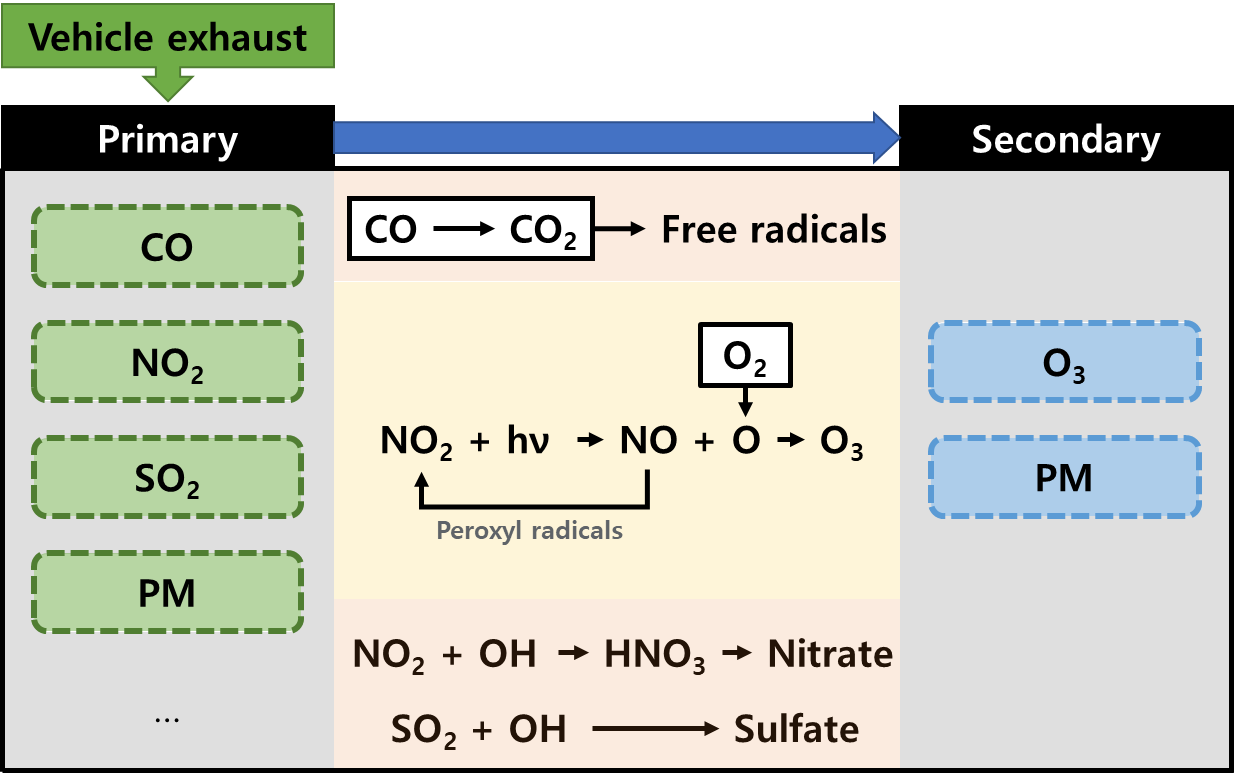
**

**Figure S1.** Secondary pollutant formation process

**Table S1.** Parameters for RF (random forest).

| **Pollutant** | **Maximum depth** | **Minimum depth** | **Minimum samples split** | **N estimators** |
| --- | --- | --- | --- | --- |
| CO | 12 | 6 | 8 | 200 |
| NO_2_ | 10 | 6 | 8 | 100 |
| O_3_ | 12 | 6 | 8 | 100 |
| PM_10_ | 12 | 6 | 8 | 200 |

**Table S2.** RNN (recurrent neural network) structure and parameters

| **Layer** | **Output Shape** | **Parameter** |
| --- | --- | --- |
| SimpleRNN | (None, 128) | 16640 |
| Dense | (None, 64) | 8256 |
| Activation | (None, 64) | 0 |
| Dense | (None, 1) | 65 |


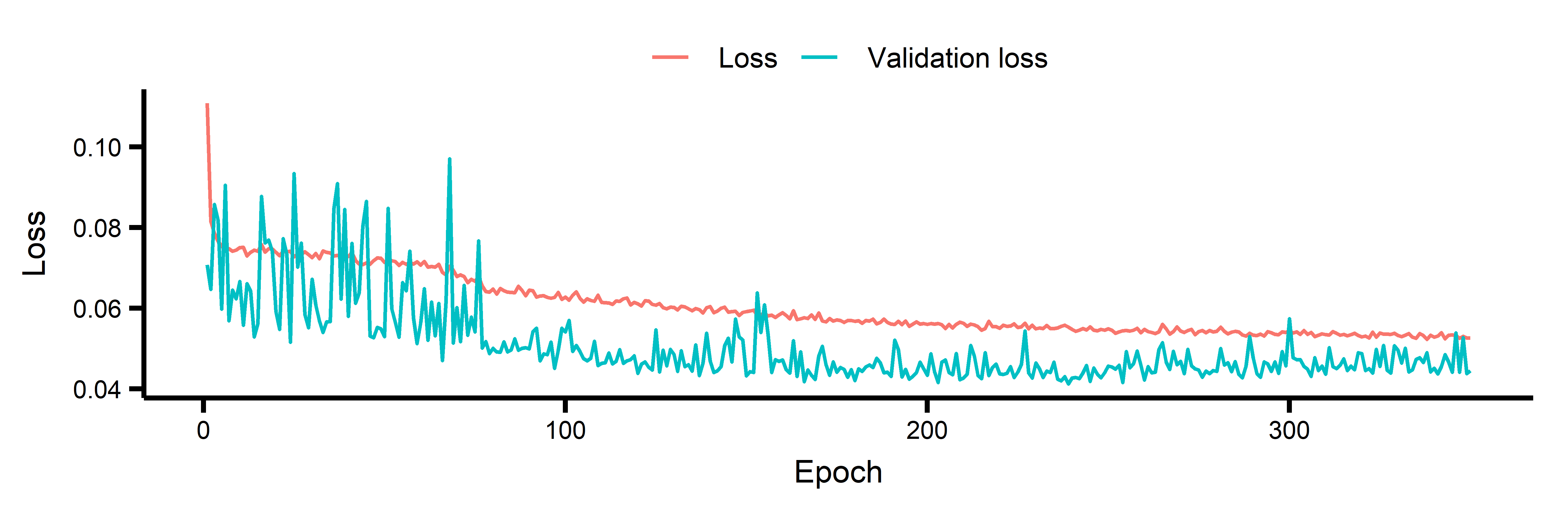


**Figure S2.** Training and validation losses over epochs in an RNN model for CO.


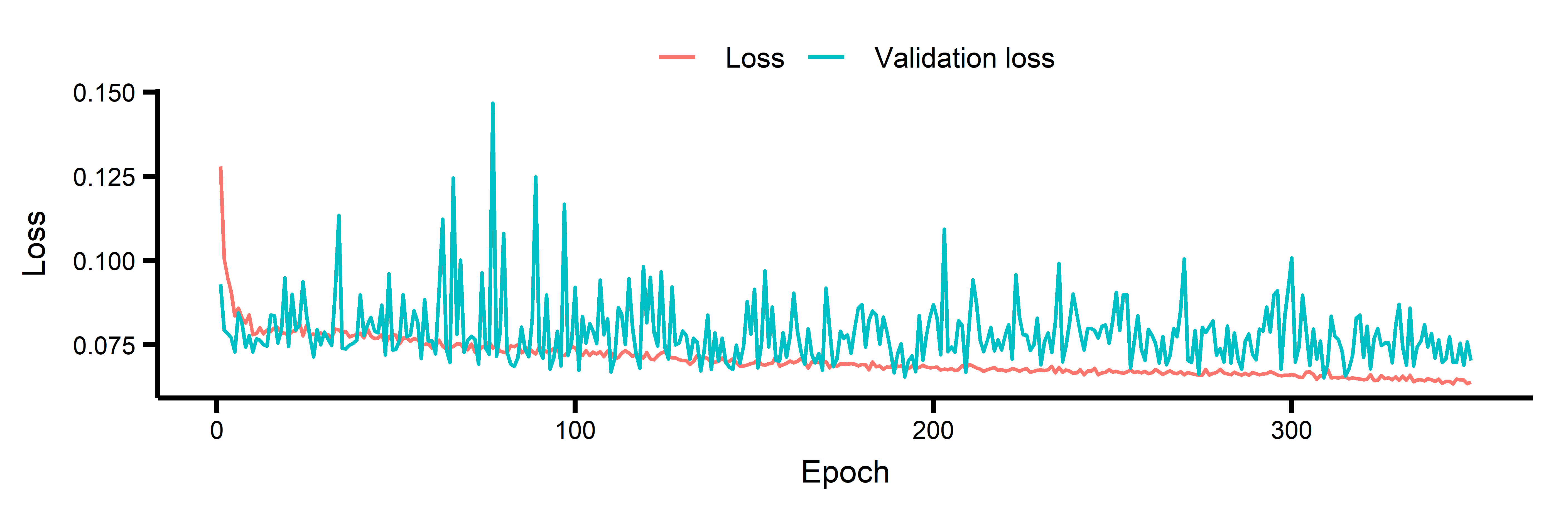


**Figure S3.** Training and validation losses over epochs in an RNN model for NO_2_.


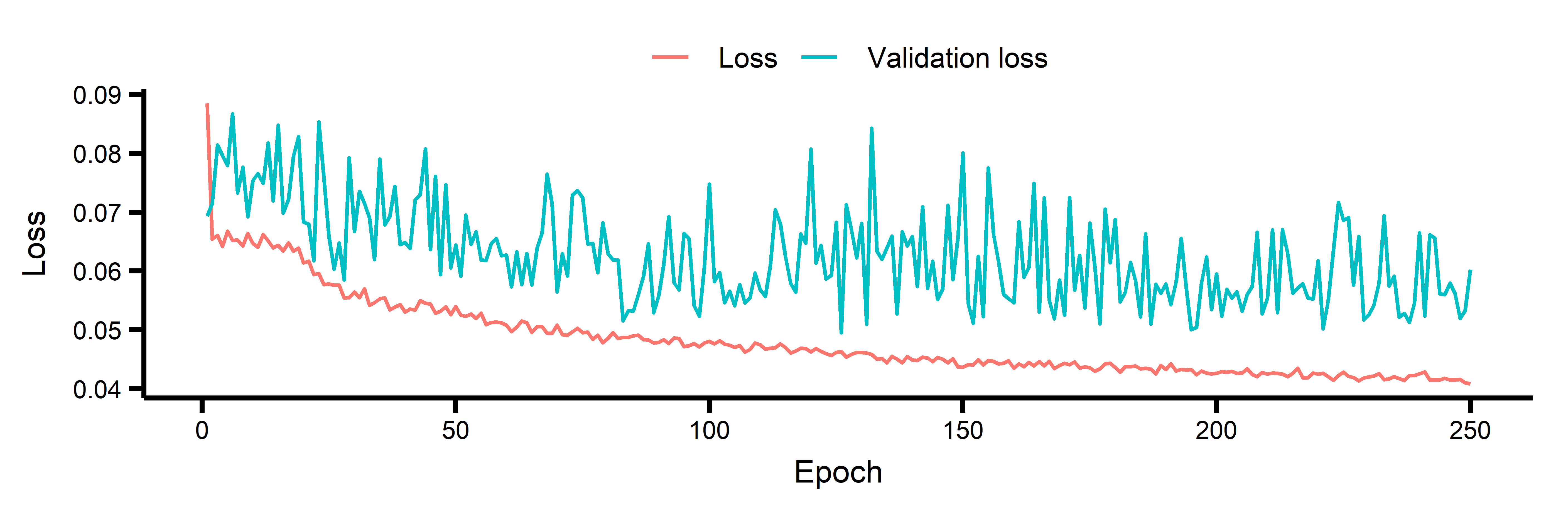


**Figure S4.** Training and validation losses over epochs in an RNN model for O_3_.


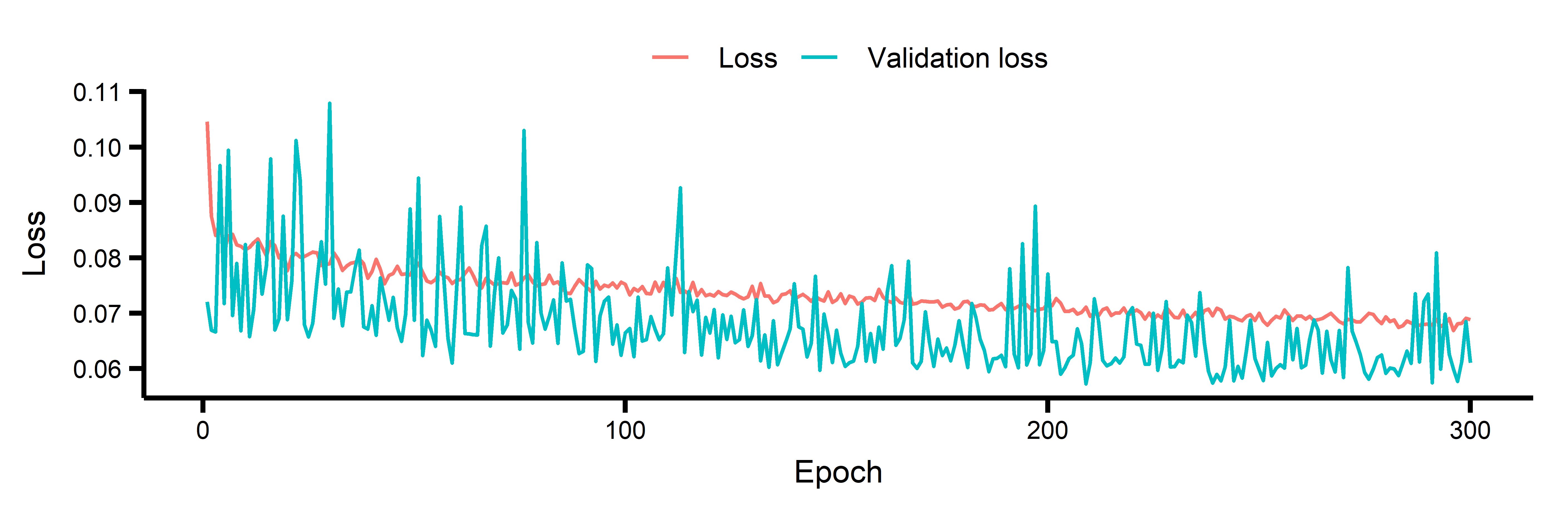


**Figure S5.** Training and validation losses over epochs in an RNN model for PM_10_.


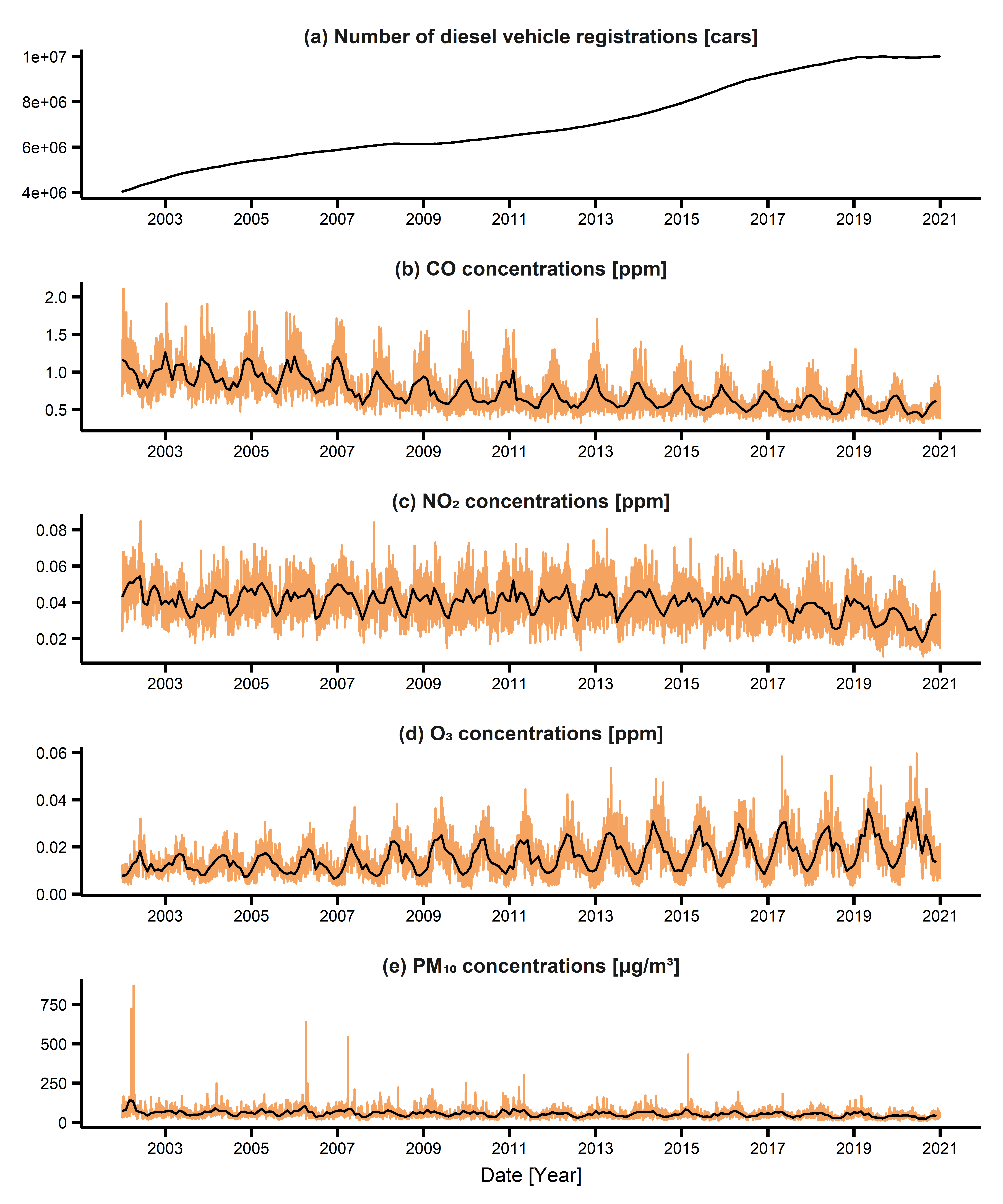


**Figure S6.** Time series graph of (a) diesel registrations, (b) CO, (c) NO_2_, (d) O_3_ and (e) PM_10_ concentrations from 2002 to 2020. The orange lines represent the daily trend, and the black lines represent the monthly trend.


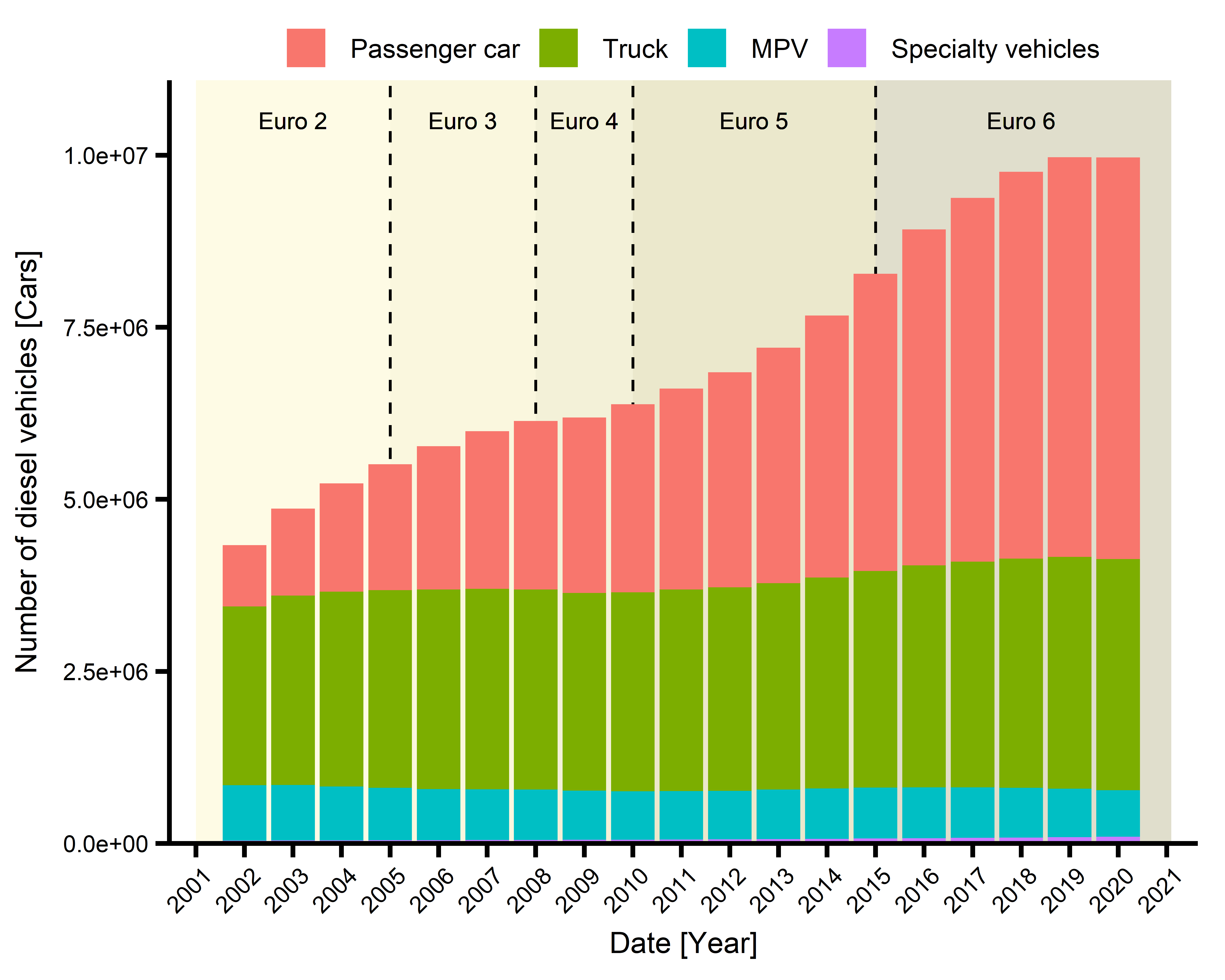


**Figure S7.** Annual number of diesel car registrations per diesel type, along with the introduction year of Euro emission standards.

**Table S3.** Result of the feature importance analysis. All units are in percentage (%).

| **Pollutant** | **CO** | **NO_2_** | **SO_2_** | **Number of diesel vehicles** | **Temperature** | **Precipitation** | **Relative humidity** | **Insolation** | **Wind (NS)** | **Wind (EW)** | **Day of year** | **Date** | **Day of week** | | | | | | |
| --- | --- | --- | --- | --- | --- | --- | --- | --- | --- | --- | --- | --- | --- | --- | --- | --- | --- | --- | --- |
|  |  |  |  |  |  |  |  |  |  |  |  |  | **MON** | **TUE** | **WED** | **THU** | **FRI** | **SAT** | **SUN** |
| CO | - | - | - | 35.8 | 14.6 | 1.0 | 1.9 | 2.4 | 22.0 | 0.5 | 6.3 | 15.1 | 0.0 | 0.0 | 0.0 | 0.0 | 0.0 | 0.0 | 0.3 |
| NO_2_ | - | - | - | 10.6 | 20.7 | 1.2 | 8.4 | 0.9 | 29.4 | 0.6 | 10.4 | 13.0 | 0.0 | 0.0 | 0.0 | 0.0 | 0.0 | 0.0 | 4.6 |
| O_3_ | 17.4 | 3.0 | 0.7 | 10.9 | 3.7 | 0.5 | 1.9 | 37.4 | 3.2 | 0.4 | 13.0 | 7.1 | 0.0 | 0.0 | 0.0 | 0.0 | 0.0 | 0.0 | 0.6 |
| PM_10_ | 12.5 | 30.6 | 32.1 | 2.8 | 3.3 | 0.6 | 3.1 | 0.7 | 5.5 | 1.7 | 4.8 | 2.2 | 0.0 | 0.0 | 0.0 | 0.0 | 0.0 | 0.0 | 0.0 |

**Table S4.** Performance of RF (random forest) and RNN (recurrent neural network) models on the validation set (2002–2015) for each air pollutant.

| **Model** | **CO** | | **NO_2_** | | **O_3_** | | **PM_10_** | |
| --- | --- | --- | --- | --- | --- | --- | --- | --- |
|  | **R^2^** | **RMSE** | **R^2^** | **RMSE** | **R^2^** | **RMSE** | **R^2^** | **RMSE** |
| RF | 0.635 | 0.135 | 0.440 | 0.006 | 0.541 | 0.004 | 0.481 | 12.119 |
| RNN | 0.746 | 0.070 | 0.634 | 0.083 | 0.759 | 0.059 | 0.694 | 0.091 |

**Table S5.** Annual observed (2002–2020), modeled (2002–2015) and predicted (2016–2020) CO concentrations.

| **Year** | **Observed** | **RF** | | **RNN** | |
| --- | --- | --- | --- | --- | --- |
|  |  | Concentrations (ppm) | Deviation (%) | Concentrations (ppm) | Deviation (%) |
| 2002 | 0.971 | 0.989 | -1.9 | 0.981 | -1.0 |
| 2003 | 1.035 | 1.014 | 2.0 | 0.978 | 5.5 |
| 2004 | 0.939 | 0.954 | -1.6 | 0.984 | -4.8 |
| 2005 | 0.957 | 0.933 | 2.5 | 0.942 | 1.6 |
| 2006 | 0.926 | 0.948 | -2.4 | 0.927 | -0.1 |
| 2007 | 0.843 | 0.821 | 2.6 | 0.835 | 0.9 |
| 2008 | 0.768 | 0.758 | 1.3 | 0.771 | -0.4 |
| 2009 | 0.732 | 0.708 | 3.3 | 0.720 | 1.6 |
| 2010 | 0.717 | 0.695 | 3.1 | 0.696 | 2.9 |
| 2011 | 0.654 | 0.675 | -3.2 | 0.683 | -4.4 |
| 2012 | 0.657 | 0.684 | -4.1 | 0.685 | -4.3 |
| 2013 | 0.649 | 0.675 | -4.0 | 0.651 | -0.3 |
| 2014 | 0.645 | 0.698 | -8.2 | 0.660 | -2.3 |
| 2015 | 0.625 | 0.697 | -11.5 | 0.636 | -1.8 |
| 2016 | 0.599 | 0.710 | -18.5 | 0.616 | -2.8 |
| 2017 | 0.569 | 0.701 | -23.2 | 0.583 | -2.5 |
| 2018 | 0.567 | 0.710 | -25.2 | 0.568 | -0.2 |
| 2019 | 0.566 | 0.724 | -27.9 | 0.561 | 0.9 |
| 2020 | 0.520 | 0.727 | -39.8 | 0.552 | -6.2 |

**Table S6.** Annual observed (2002–2020), modeled (2002–2015) and predicted (2016–2020) NO_2_ concentrations.

| **Year** | **Observed** | **RF** | | **RNN** | |
| --- | --- | --- | --- | --- | --- |
|  |  | Concentrations (ppm) | Deviation (%) | Concentrations (ppm) | Deviation (%) |
| 2002 | 0.046 | 0.045 | 2.2 | 0.044 | 4.3 |
| 2003 | 0.038 | 0.041 | -7.9 | 0.040 | -5.3 |
| 2004 | 0.043 | 0.042 | 2.3 | 0.042 | 2.3 |
| 2005 | 0.044 | 0.042 | 4.5 | 0.041 | 6.8 |
| 2006 | 0.042 | 0.042 | 0.0 | 0.041 | 2.4 |
| 2007 | 0.043 | 0.042 | 2.3 | 0.041 | 4.7 |
| 2008 | 0.041 | 0.041 | 0.0 | 0.041 | 0.0 |
| 2009 | 0.041 | 0.041 | 0.0 | 0.040 | 2.4 |
| 2010 | 0.043 | 0.041 | 4.7 | 0.040 | 7.0 |
| 2011 | 0.041 | 0.041 | 0.0 | 0.039 | 4.9 |
| 2012 | 0.041 | 0.042 | -2.4 | 0.040 | 2.4 |
| 2013 | 0.041 | 0.041 | 0.0 | 0.040 | 2.4 |
| 2014 | 0.041 | 0.043 | -4.9 | 0.041 | 0.0 |
| 2015 | 0.040 | 0.044 | -10.0 | 0.041 | -2.5 |
| 2016 | 0.040 | 0.043 | -7.5 | 0.039 | 2.5 |
| 2017 | 0.037 | 0.043 | -16.2 | 0.038 | -2.7 |
| 2018 | 0.035 | 0.043 | -22.9 | 0.037 | -5.7 |
| 2019 | 0.034 | 0.045 | -32.4 | 0.037 | -8.8 |
| 2020 | 0.028 | 0.044 | -57.1 | 0.036 | -28.6 |

**Table S7.** Annual observed (2002–2020), modeled (2002–2015) and predicted (2016–2020) O_3_ concentrations.

| **Year** | **Observed** | **RF** | | **RNN** | |
| --- | --- | --- | --- | --- | --- |
|  |  | Concentrations (ppm) | Deviation (%) | Concentrations (ppm) | Deviation (%) |
| 2002 | 0.012 | 0.012 | 0.0 | 0.012 | 0.0 |
| 2003 | 0.012 | 0.011 | 8.3 | 0.012 | 0.0 |
| 2004 | 0.012 | 0.012 | 0.0 | 0.012 | 0.0 |
| 2005 | 0.013 | 0.013 | 0.0 | 0.013 | 0.0 |
| 2006 | 0.012 | 0.012 | 0.0 | 0.013 | -8.3 |
| 2007 | 0.012 | 0.013 | -8.3 | 0.013 | -8.3 |
| 2008 | 0.015 | 0.014 | 6.7 | 0.014 | 6.7 |
| 2009 | 0.016 | 0.016 | 0.0 | 0.015 | 6.3 |
| 2010 | 0.015 | 0.015 | 0.0 | 0.015 | 0.0 |
| 2011 | 0.015 | 0.015 | 0.0 | 0.016 | -6.7 |
| 2012 | 0.016 | 0.016 | 0.0 | 0.016 | 0.0 |
| 2013 | 0.018 | 0.016 | 11.1 | 0.018 | 0.0 |
| 2014 | 0.018 | 0.016 | 11.1 | 0.019 | -5.6 |
| 2015 | 0.017 | 0.016 | 5.9 | 0.020 | -17.6 |
| 2016 | 0.018 | 0.016 | 11.1 | 0.019 | -5.6 |
| 2017 | 0.019 | 0.016 | 15.8 | 0.020 | -5.3 |
| 2018 | 0.019 | 0.016 | 15.8 | 0.021 | -10.5 |
| 2019 | 0.021 | 0.016 | 23.8 | 0.021 | 0.0 |
| 2020 | 0.023 | 0.016 | 30.4 | 0.023 | 0.0 |

**Table S8.** Annual observed (2002–2020), modeled (2002–2015) and predicted (2016–2020) PM_10_ concentrations.

| **Year** | **Observed** | **RF** | | **RNN** | |
| --- | --- | --- | --- | --- | --- |
|  |  | Concentrations (ppm) | Deviation (%) | Concentrations (ppm) | Deviation (%) |
| 2002 | 65.5 | 64.4 | 1.6 | 59.8 | 8.6 |
| 2003 | 58.0 | 59.4 | -2.6 | 57.1 | 1.4 |
| 2004 | 60.9 | 61.6 | -1.2 | 58.4 | 4.2 |
| 2005 | 57.1 | 59.0 | -3.4 | 57.3 | -0.3 |
| 2006 | 62.6 | 61.5 | 1.7 | 61.0 | 2.5 |
| 2007 | 58.0 | 59.4 | -2.5 | 57.0 | 1.6 |
| 2008 | 50.7 | 52.8 | -4.0 | 50.6 | 0.3 |
| 2009 | 51.6 | 51.3 | 0.6 | 49.4 | 4.3 |
| 2010 | 54.5 | 52.6 | 3.5 | 51.9 | 4.8 |
| 2011 | 51.8 | 50.3 | 2.9 | 48.0 | 7.4 |
| 2012 | 48.5 | 49.6 | -2.3 | 49.5 | -2.1 |
| 2013 | 48.9 | 51.6 | -5.5 | 49.8 | -1.9 |
| 2014 | 53.6 | 52.9 | 1.3 | 53.2 | 0.8 |
| 2015 | 49.7 | 50.4 | -1.4 | 51.2 | -3.0 |
| 2016 | 52.8 | 48.3 | 8.5 | 50.4 | 4.4 |
| 2017 | 51.4 | 45.7 | 11.1 | 50.3 | 2.2 |
| 2018 | 46.6 | 41.0 | 12.1 | 51.1 | -9.7 |
| 2019 | 45.4 | 41.1 | 9.5 | 53.8 | -18.6 |
| 2020 | 37.5 | 32.4 | 13.8 | 48.5 | -29.3 |
